# Phacoemulsification Combined with Pars Plana Vitrectomy: Outcome in Horses with Acquired Cataracts Associated with Uveitis

**DOI:** 10.3390/ani14081192

**Published:** 2024-04-16

**Authors:** Andrey Kalinovskiy, Stephan Leser, Anna Ehrle, Sven Reese, Sara Jones, Hartmut Gerhards

**Affiliations:** 1 Hanse Equine Clinic, 27419 Sittensen, Germany; 2 Equine Clinic, Surgery and Radiology, Free-University of Berlin, 14195 Berlin, Germany; 3 Veterinary Department, Institute of Veterinary Anatomy, Histology and Embryology, Ludwig-Maximilians-University Munich, 80539 Munich, Germany; s.reese@anat.vetmed.uni-muenchen.de; 4 Independent Researcher, 80539 Munich, Germany

**Keywords:** cataract, chronic uveitis, equine, phacoemulsification, pars plana vitrectomy, retinal detachment

## Abstract

**Simple Summary:**

Uveitis in horses is the most common ocular disease and causes blindness and cataracts in horses. The aim of this study was to evaluate vision and complications after phacoemulsification (emulsification and aspiration of the lens cortex/nucleus from the eye) combined with pars plana vitrectomy (removal of vitreous body from the vitreous chamber) in horses with uveitis-associated cataracts. Additionally, the surgical technique with and without posterior capsulorhexis (removal of the posterior lens capsule) during phacoemulsification (PE) was compared. Uveitis-associated cataracts were treated with phacoemulsification combined with pars plana vitrectomy under identical conditions in 12 eyes of 10 horses with posterior capsulorhexis and 20 eyes of 18 horses without posterior capsulorhexis. Horses underwent pre-, postoperative, and follow-up examinations. In the period 12–18 months postoperatively, 3/7 (42.9%) eyes treated without posterior capsulorhexis, and 2/9 (22.2%) eyes treated with posterior capsulorhexis were visual. In the period > 24 months postoperatively, 2/7 (28.6%) eyes treated without posterior capsulorhexis and 1/8 (12.5%) eyes treated with posterior capsulorhexis were visual. In the overall population, a significant decrease in the number of eyes with postoperative active uveitis was observed during follow-up examinations (*p* < 0.001). A significant increase in the number of eyes that were blind due to retinal detachment was observed in the overall patient population as the examination period progressed (*p* < 0.001). Retinal detachment was the sole long-term cause of blindness. In horses diagnosed with uveitis-associated cataracts, no persistent active uveitis was observed following the described treatment during the follow-up examinations. However, the proportion of eyes that were blind due to retinal detachment increased. Thus, while the surgery may prevent postsurgical persistent active uveitis and remove lens opacity, the prognosis for a visual outcome is guarded. A superior outcome in postsurgical vision was observed in eyes treated without posterior capsulorhexis.

**Abstract:**

Background: Cataracts resulting from equine recurrent uveitis (ERU) or other forms of uveitis are usually associated with rapid progression. ERU is the most common ocular disease cause of blindness and cause of cataracts in horses. The necessity for the posterior capsulorhexis (PC) during phacoemulsification (PE) is controversial. This study aimed to evaluate vision and complications after PE combined with pars plana vitrectomy (PPV) in horses with uveitis-associated cataracts and compare the PE technique with and without posterior capsulorhexis. Methods: Thirty-two eyes of 28 horses with uveitis-associated cataracts aged 14 months to 19.6 years were treated with PE-PPV under identical conditions. Twenty-three eyes of 21 horses were affected by an ERU-associated (ERU group), and nine eyes of 7 horses were affected by cataracts related to uveitis with pathogenesis different to ERU (non-ERU group). PE-PPV was performed in 12 eyes of 10 horses (PC group) and 20 eyes of 18 horses without posterior capsulorhexis (NPC group). Follow-up examination was performed at a mean of 1.7 ± 1.8 years postoperatively (range: 1 month–6.4 years). Results: In the period up to 1 month postoperatively, 17/20 (85%) NPC-eyes and 8/12 (67%) PC-eyes (total: 25/32 [78%]) were visual. From 1–6 months postoperatively, 16/20 (80%) NPC-eyes and 7/12 (58.3%) PC-eyes (total: 23/32 [72%]), and from 6–12 months, 7/11 (63.6%) NPC-eyes and 3/8 (37.5%) PC-eyes (total: 10/19 [52.6%]) were visual. From 12–18 months postoperatively, 3/7 (42.9%) NPC-eyes and 2/9 (22.2%) PC-eyes (total: 5/16 [31.3%]), and from 18–24 months, 3/8 (37.5%) NPC-eyes and 1/8 (12.5%) PC-eyes (total: 4/16 [25%]) were visual. After 24 months postoperatively, 2/7 (28.6%) NPC-eyes and 1/8 (12.5%) PC-eyes (total: 3/15 [20%]) were visual. Despite the higher number of visual eyes in the NPC group at each time point, differences were not significant. No obvious differences regarding postsurgical vision were observed between the ERU- and non-ERU groups at each time point. In the overall population, a significant decrease in the number of eyes with postoperative active uveitis was observed during the follow-up examinations (*p* < 0.001). A significant increase in the number of eyes that were blind due to retinal detachment was observed in the overall patient population as the examination period progressed (*p* < 0.001). Retinal detachment was the sole long-term cause of blindness. Conclusions: In horses diagnosed with uveitis-associated cataracts and treated with PE-PPV, no persistent active uveitis was observed in the present study during follow-up examinations. However, the proportion of eyes that were blind due to retinal detachment increased. Whilst PE-PPV may prevent postsurgical persistent active uveitis and remove lens opacity, the prognosis for a visual outcome is guarded. A superior outcome in postsurgical vision was observed in the NPC group. However, caution is required when interpreting these results due to several factors that affect the independent comparison of the surgical groups.

## 1. Introduction

Approximately 5–7% of all horses with otherwise healthy eyes are estimated to have a cataract [1]. Cataracts resulting from equine recurrent uveitis (ERU) or other forms of uveitis are usually associated with rapid progression [1]. In adult horses, ERU is the most common ocular disease, cause of blindness, and the most common cause of acquired cataracts [2,3]. In the pathogenesis of ERU, autoimmune inflammatory responses and leptospiral-related intraocular infection may play a role [4,5]. Besides cataracts, glaucoma, degeneration of the corneal endothelium, phthisis bulbi and retinal detachment (RD) are among the long-term sequelae of ERU or other forms of uveitis [4].

Currently, no conservative treatment options exist in case of complete cataract impairing vision, and the only true therapy is surgical removal [1]. Phacoemulsification (PE), where the lens cortex/nucleus is emulsified using ultrasound and aspirated from the eye, is currently considered the treatment of choice for all types of cataracts affecting vision [6,7]. It is generally recommended to consider this treatment option only in the absence of concurrent systemic or ophthalmic disease [1]. The postoperative prognosis for PE in uveitis-associated cataracts is guarded [6,8]. However, some studies [1,7,9] suggest that PE remains the treatment of choice when concomitant degenerative diseases (especially retinal diseases) and acute inflammation are ruled out and when the uveitis is under medical control or has been treated with pars plana vitrectomy (PPV; removal of vitreous body from the vitreous chamber) or a cyclosporine-A-releasing implant.

In human ophthalmology, PE-PPV is the preferred method for the management of uveitis-associated cataracts, and the combination of both surgeries has also been described in equine ophthalmology to prevent postoperative uveitis [9,10]. Some studies recommend PPV only in case of a positive ocular leptospiral test [5,11]. Other authors consider PPV also indicated in the case of previous uveitis with accumulation of inflammatory products or lens remnants in the vitreous [12,13,14].

Removal of the posterior lens capsule (PC; posterior capsulorhexis) has been recommended if it is opacified, traumatised intraoperatively, or to prevent postoperative capsular opacification (PCO) in the posterior lens capsule [8,9]. However, human and equine ophthalmology studies have reported an increased risk of RD following this procedure [15,16].

The most important intraoperative complications of PE include posterior lens capsule tear, retinal folds and RD [6,17]. Persistent active uveitis, corneal oedema/ulceration, PCO in the posterior lens capsule, glaucoma and RD are the most important postoperative PE complications [6,7,9]. The most relevant postoperative PPV complications include lens trauma, vitreous/retinal haemorrhage, and RD [14,18].

Uveitis-associated cataract is a particular challenge for the equine ophthalmologist. In this case, both the cataract and the uveitis must be treated, and the incidence of postsurgical persistent active uveitis is high [6,7,19]. The aim of this study was to assess the intraoperative course and postsurgical outcome of PE-PPV for the management of uveitis-associated cataracts in 32 eyes of 28 horses. To the best of the authors’ knowledge, this topic has not been evaluated previously. Additionally, the comparison of surgical methods, including PE with and without posterior capsulorhexis, is lacking to date. The authors hypothesised that posterior capsulorhexis would be associated with an increased risk of intra- and postoperative complications. It was further suspected that in the overall patient population, the incidence of postoperative persistent active uveitis would be low.

## 2. Materials and Methods

### 2.1. Patient Selection

**Figure 1:**
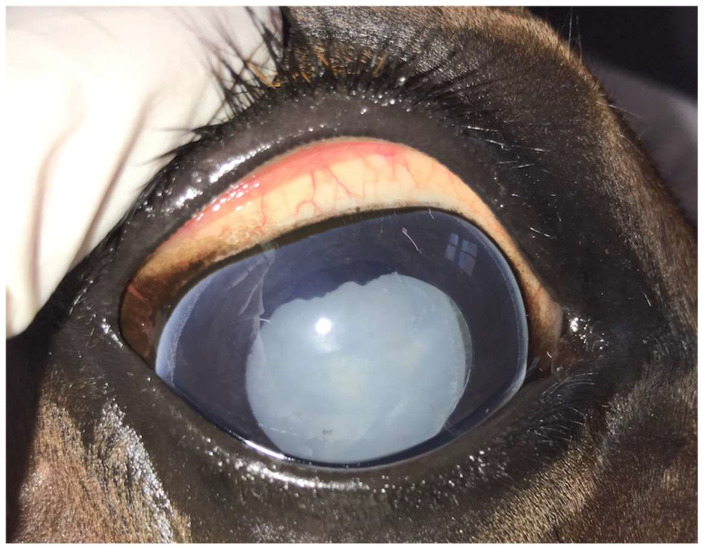
Mature uveitis-associated cataract in the right eye of a 12-year-old Warmblood mare.

Medical records of two referral hospitals (Hanse equine clinic, Sittensen, Germany, and Grosbois equine clinic, Marolles-en-Brie, France) were retrospectively reviewed for horses undergoing PE-PPV for treatment of uveitis-associated cataracts between 2013 and 2020. The inclusion criteria for this study were: same surgeon (S.L.), surgical technique, equipment, anaesthetic protocol; no topical or systemic medication at the time of presentation; quiescent (non-active) phase of uveitis at the time of presentation; no other surgeries on the affected eye; horses over 12 months of age; cataracts with a previous history of uveitis; PPV and PE performed under one general anaesthesia; at least one follow-up examination ≥1 month postoperatively. ERU-associated lens opacities and lens opacities related to uveitis with pathogenesis different to ERU (non-ERU) are both classified as uveitis-associated cataracts throughout this study [13,20,21]. The diagnosis was based on history, ophthalmic and ultrasonographic examination (characteristic ophthalmic, ultrasonographic changes and SPOTS Ocular-Scoring-System) (Figure 1) [3,14,22]. Vitreous debris associated with vitreous degeneration was identified during the presurgical ultrasonographic examination, and the vitreous appearance intraoperatively was identified by direct visualisation after PE and before PPV. ERU-associated cataract (ERU group) was diagnosed if there was a history of recurrent inflammatory episodes with a minimum of two episodes reported. Non-ERU-associated cataract (non-ERU group) was diagnosed if there was no history of uveitis recurrence (one uveitis episode only). In addition, an effort was made to support the correct assignment into the ERU- or non-ERU group by performing the rapid leptospiral test intraoperatively.

For accurate differentiation of cataract maturity, cataracts were divided into immature, mature, or hypermature in this study [21]. The exclusion criteria for this study were diseases associated with blindness or increased risk of complications resulting in poor postoperative vision (particularly retinal diseases, corneal opacities, keratitis, phthisis bulbi, lens luxation/subluxation); resorbing cataracts or incipient and incomplete cataracts without clinically relevant visual impairment according to SPOTS Ocular Scoring System [22]; concomitant systemic disease and temperament that would not tolerate perioperative medication. In total, 53 eyes of 45 horses underwent PE-PPV between 2013 and 2020. Of these, 32 eyes (16 left and 16 right eyes) of 28 horses met the inclusion criteria of this study. Four out of twenty-eight horses underwent PE-PPV in both eyes, where the second surgery was performed at least one month after the initial surgery.

### 2.2. Preoperative Examination

A physical examination and evaluation of vision (menace response, maze test) were performed. Further examination included evaluation of the pupillary reflex and dazzle reflex, intraocular pressure measurement (Tono-Pen AVIA-VET, Reichert Technologies, AMETEK, Muenchen, Germany), direct ophthalmoscopy (Heine Beta-200, HEINE Optotechnik, Gilching, Germany), indirect ophthalmoscopy with 20D lens (Heine Omega-500-LED, HEINE Optotechnik, Gilching, Germany), slit-lamp biomicroscopy (Kowa SL-14, Kowa Company, Tokyo, Japan) and sonography (MyLabEightVET, ESAOTE, Koeln, Germany) (*n* = 28).

### 2.3. PE-PPV

As part of a standard presurgical protocol four days prior to surgery, affected eyes were treated topically three times daily with both atropine eye drops (Atropin-POS-0.5%, URSAPHARM, Saarbruecken, Germany) for appropriate mydriasis and dexamethasone, neomycin-sulphate and polymyxin-B-sulphate containing ophthalmic ointment (Isopto-Max, Novartis-Pharma, Nuernberg, Germany). Systemically, the horses received Flunixin-meglumine 1.1 mg/kg intravenously (Flunidol, CP-Pharma Handelsgesellschaft, Burgdorf, Germany).

Thirty minutes before surgery, acepromazine 0.5 mg/kg (Tranquisol P, CP-Pharma Handelsgesellschaft, Burgdorf, Germany) was administered intramuscularly. Xylazine 0.08 mg/kg (Xylavet, CP-Pharma Handelsgesellschaft, Burgdorf, Germany), levomethadone 0.05 mg/kg (L-Polamivet, Intervet, Unterschleißheim, Germany) and flunixin-meglumine 1.1 mg/kg (Flunidol, CP-Pharma Handelsgesellschaft, Burgdorf, Germany) were administered intravenously right before induction. General anaesthesia was induced intravenously with diazepam 0.05 mg/kg (Ziapam, Laboratoire TVM, Lempdes, France) and ketamine 2.5 mg/kg (Ketamin, CP-Pharma Handelsgesellschaft, Burgdorf, Germany). Horses were subsequently intubated and positioned in left or right lateral recumbency, depending on the affected eye. The periocular region was prepared for surgery using standard methods [19]. During general anaesthesia, the end-tidal isoflurane concentration (FE’Iso) (Isofluran-CP 1 mg/mL, CP-Pharma Handelsgesellschaft, Burgdorf, Germany) was set at approximately 3.5–4.5% depending on the depth of anaesthesia. Additionally, a balanced crystalline solution infusion 10 mL/kg/h (Ringer-Lactat Hartmann B. Braun-Vet-Care, Braun, Melsungen, Germany) and xylazine (Xylavet, CP-Pharma Handelsgesellschaft, Burgdorf, Germany) continuous rate infusion 0.8 mg/kg/h were administered for the duration of anaesthesia. Ketamine 1.1 mg/kg (Ketamin; CP-Pharma Handelsgesellschaft, Burgdorf, Germany) was administered intravenously immediately prior to approach creation to avoid inappropriate tension or movement of the globe during surgery. A bulbus rotator for horses (An-vision, Salt Lake City, UT, USA) was used for intraoperative manipulation of the eye. At first, an approach for the intravitreal infusion tube for the PPV coupled to the vitrectomy device (Phakotom E and Aspimat E, Erbe, Elektromedizin, Tuebingen, Germany) was made at the 11 o’clock position, 14 mm above the limbus with a CO_2_-Laser (12-Watt, single mode, Smartxide, DEKA M.E.L.A., Calenzano, Italy) and subsequently dilatated with a trocar. This ensured that the intraocular pressure (IOP) was continuously maintained at 25 mmHg during surgery. All the following PE steps were carried out under magnification (Surgical-Microscope-Advanced, Eickemeyer, Tuttlingen, Germany). An amount of 2 mL of aqueous humor was aspirated from the anterior chamber (AC) using limbal paracentesis, and 2 mL hyaluronic acid (Sodium hyaluronate 2.2%-2 mL, An-vision, Salt Lake City, UT, USA) was injected. The aqueous humor was examined with a rapid leptospiral test during surgery (SNAP Lepto-Test, IDEXX Laboratories, Westbrook, ME, USA) [23]. A limbus-based, self-sealing, 3 mm-long incision into the AC was created. A sclerocorneal U-suture (6-0 USP, Polyglactin 910, Vicryl, Ethicon Inc., Somerville, MA, USA) was then pre-placed lateral to the incision [9]. Anterior capsulorhexis was performed using a cystotome (23-gauge), and hyaluronic acid was injected into the subcapsular to separate the lens cortex from the capsule. The PE-titanium needle (angle: 30°, length: 39 mm, diameter: 0.9 mm) in sleeve (length: 24 mm, diameter: 1.1 mm) (Oertli titano hexadisq, Oertli Instrumente, Berneck, Switzerland) was inserted through the incision into the AC and then into the lens. The lens cortex/nucleus was emulsified and aspirated (PE-unit: CataRhex, Oertli Instrumente, Berneck, Switzerland).

Additionally, posterior capsulorhexis was performed in 12 eyes. Posterior capsulorhexis was added in cases where there was tearing or opacification of the posterior lens capsule and failure of separation of the lens cortex from the posterior lens capsule. Based on this surgical step, the surgical group-NPC (no posterior capsulorhexis) and surgical group-PC (posterior capsulorhexis) were defined. Posterior capsulorhexis was performed as small as possible to preserve the physiological separation between the anterior and vitreous chambers. Finally, the limbus-based scleral incision was closed with the pre-placed U-suture, and the conjunctiva was apposed in a simple continuous pattern (6-0 USP, Polyglactin 910, Vicryl, Ethicon Inc., Somerville, MA, USA).

**Figure 2:**
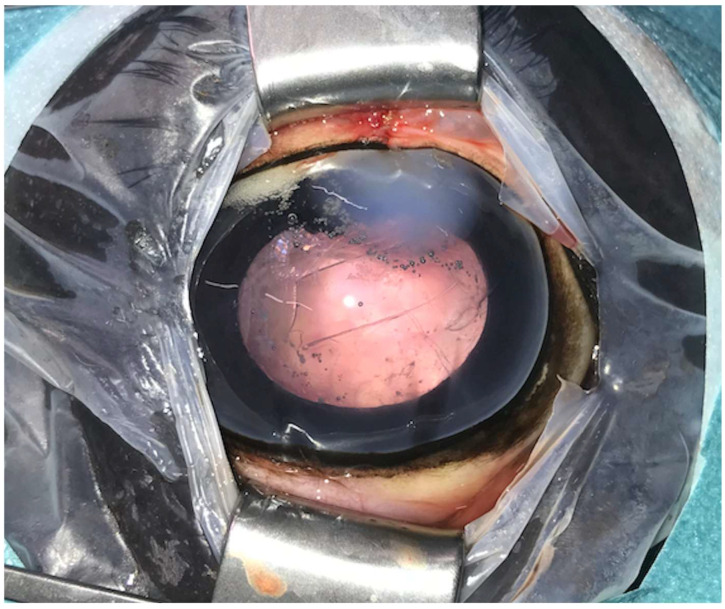
Postoperative image immediately after phacoemulsification combined with pars plana vitrectomy with the posterior lens capsule preserved (same eye as in Figure 1). Postoperative corneal oedema is evident at the one o’clock position (approach region). The anterior capsulorhexis is evident centrally.

The PPV was performed as previously described [24]. The approach for the customised vitrectomy handpiece (length: 60 mm, diameter: 1.8 mm) was created at the one o’clock position, similar to the intravitreal infusion approach. The vitreous body was removed (settings: magnetic powered cutting mechanism [600 cuts/min], max. aspiration vacuum of 480 mm Hg and flow rate of 30 mL/min; vitrectomy device: Phakotom E and Aspimat E, Erbe, Elektromedizin, Tuebingen, Germany) under transpupillary control (indirect binocular ophthalmoscope: Heine Omega 500 LED, HEINE Optotechnik, Gilching, Germany) and replaced with balanced salt solution (250 mL mixed with 20 mg gentamicin and 0.5 mg epinephrin) positioned 110 cm above the treated eye. Care was taken to remove >90% of the vitreous [14,25]. Finally, the Tenon’s capsule and the conjunctiva were closed in a simple continuous pattern as described above. At last, subconjunctival injection of 20 mg gentamicin (Gentamicin-ratiopharm, 40 mg/mL, Ratiopharm, Ulm, Germany) and dexamethasone (Dexa-ratiopharm, 40 mg/mL, Ratiopharm, Ulm, Germany) each was performed (Figure 2). The preoperatively topical medication was continued for 14 days, and systemic treatment for 7 days post-surgery.

### 2.4. Postoperative Examination

In addition to the daily routine monitoring, an extended postoperative examination was performed on 28 horses and included all the steps described for the preoperative examination.

### 2.5. Follow-Up Examination

A follow-up examination was performed on 25 horses. The examination was similar to the preoperative protocol. Ultrasonographic examination was included in 19 horses. During the follow-up examination, the owners were questioned regarding the recurrence rate of the uveitis after the surgery.

### 2.6. Data Analysis

Data were analysed using IBM SPSS Statistics 26 (SPSS Inc., an IBM Company, Chicago, IL, USA). The data were assessed for normality using the Shapiro–Wilk test and Kolmogorov-Smirnov test. The ordinally and nominally distributed variables were tested for dependency using the Chi-square test. The effective measure of association phi was calculated. Where the expected frequencies were lower than 5, Fisher’s exact test was used. The results of the three primary factors influencing postoperative vision (surgical technique, cataract aetiology, and horse’s age at the time of surgery) were tested for correlation using binary logistic regression. The odds ratio (OD) was calculated as the effect measure. Values with *p* < 0.05 were considered significant.

In order to evaluate postoperative ophthalmic findings and identify the overall number of visual eyes at the last follow-up, examination times were divided into preoperative, 1–14 days postoperative and ≥1 month postoperative (follow-up examination). If more than one follow-up examination was performed, the last available examination was used. Additionally, the follow-up examinations (≥1 month postoperatively) assessing postoperative vision were divided into the following six time periods: <1 month, 1–6 months, 6–12 months, 12–18 months, 18–24 months, and >24 months. No eye was examined more than once in each period.

## 3. Results

Thirty-two eyes of twenty-eight horses with uveitis-associated cataracts were included in the study. The horses’ breed and sex distribution are shown in Table 1. A total of 23 eyes of 21 horses were affected by an ERU-associated and 9 eyes of 7 horses by non-ERU-associated cataract. The rapid leptospiral test was performed in 29/32 eyes. The test was positive in 12/20 eyes (60%) with ERU-associated cataracts (total 12/29 [41%]). None of the eyes with non-ERU-associated cataracts tested positive (*n* = 9) (*p* = 0.003). PE-PPV was performed in 12 eyes of 10 horses with posterior capsulorhexis (PC group) and in 20 eyes of 18 horses without posterior capsulorhexis (NPC group). There were 5 eyes (25%) with non-ERU and 15 eyes (75%) with ERU-associated cataracts in the NPC group and 4 eyes (33%) with non-ERU and 8 eyes (67%) with ERU-associated cataracts in the PC group (*p* = 0.696). A non-significant difference between the surgical groups was observed in both the localization and maturity of the cataract. Advanced cataract stages occurred more frequently in the PC group (Table 2).

The mean age in the overall patient population was 8 ± 4.1 years (range: 14 months–19.6 years). The mean age in the NPC group was 8 ± 2.8 years (range: 3.7–12.3 years), and in the PC group, 8.6 ± 5.8 years (range: 14 months–19.6 years). Horses with ERU-associated cataracts had a mean age of 8.7 ± 4.1 years (range: 1.2–19.6 years), and horses with non-ERU-associated cataracts 6.5 ± 4.5 years (range: 1.5–10.2 years). There were no significant differences in age between the surgical and aetiological (ERU- or non-ERU) groups.

The preoperative examination was performed in 32 eyes of 28 horses. The postoperative examination was performed in 32 eyes of 28 horses 7 ± 3.7 days (range: 1–14 days) post-surgery. A follow-up examination was performed on 28 eyes of 25 horses. In 10 eyes of 7 horses, a follow-up examination was conducted more than once. The follow-up examination was performed at 1.7 ± 1.8 years (range: 1 month–6.4 years) postoperatively. In 4 eyes of 4 horses, follow-up was not available. Ultrasonography was included in 21 eyes of 19 horses.

### 3.1. Intraoperative Findings

The intraoperative findings are summarised in Table 2. Vitreous debris associated with vitreous degeneration was detected in the overall patient population. The number of eyes with intraoperatively observed retinal folds (*p* = 0.002), vitreous haemorrhage (*p* = 0.044) and displacement of lens remnants into the vitreous body (*p* = 0.044) was significantly higher in the PC group. Intraoperative retinal detachment occurred non-significantly more often in the PC group. In two out of three cases of vitreous haemorrhage, this was associated with concurrent retinal detachment. No obvious differences were observed between aetiological groups.

### 3.2. Dynamics of Ophthalmic Changes

The presurgical and postsurgical ophthalmic findings are summarised in Table 3 and Table 4. No AC-flare was observed preoperatively in the overall patient population; however, on examination 1–14 days postoperatively, the number of eyes with AC-flare was significantly higher in the PC group compared to the NCP group (*p* = 0.008). The number of eyes with hyperechogenic material in the vitreous chamber was non-significantly higher in the ERU group preoperatively, and no obvious differences were seen on examinations of 1–14 days and ≥1 month postoperatively. Diffuse corneal oedema occurred non-significantly more often in the PC group on examination 1–14 days and ≥1 month postoperatively. Postoperative hypertension on examination 1–14 days postoperatively occurred in the ERU group only; however, the difference was not significant. The number of eyes with posterior synechia was significantly higher in the ERU group (*p* = 0.035) on presurgical examination and non-significantly higher on examination 1–14 days and ≥1 month postoperatively. Despite the higher number of eyes with dyscoria in the ERU group preoperatively, examination 1–14 days and ≥1 month postoperatively, differences were not significant. The number of eyes with vitreous chamber haze, lens remnants in the vitreous chamber and postoperative active uveitis was non-significantly higher in the PC group on examination 1–14 days postoperatively. PCO in the posterior lens capsule occurred non-significantly more often in the ERU group on examination 1–14 days and ≥1 month postoperatively. The number of eyes with RD was non-significantly higher in the PC group on examination 1–14 days and significantly higher on examination ≥ 1 month (*p* = 0.001) postoperatively. Regarding the number of eyes with posterior synechia, dyscoria, hyperechogenic material in the vitreous chamber, postoperative hypertension, as well as retinal fold formation and retinal oedema, no obvious differences were observed between surgical groups during any examination period. Additionally, no obvious differences were observed in the number of eyes with AC-flare, diffuse corneal oedema, lens remnants in the vitreous chamber, vitreous chamber haze, postoperative active uveitis, RD as well as retinal fold formation and retinal oedema between aetiological groups during any examination period.

A significant decrease in the number of eyes with diffuse corneal oedema (*p* = 0.038), AC-flare (*p* < 0.001), vitreous chamber haze (*p* < 0.001) as well as in hyperechogenic material in the posterior chamber (*p* < 0.001) and postoperative active uveitis (*p* < 0.001) was detected in the overall patient population when comparing the examinations 1–14 days and ≥1 month postoperatively. Additionally, no postsurgical episodes of uveitis recurrence were reported by the owners at the time of the examination ≥ 1 month postoperatively. The number of cases with PCO in the posterior lens capsule (evaluated in the NPC group only) increased significantly when the examination 1–14 days and ≥1 month postoperatively were compared (*p* = 0.027). The number of eyes with RD increased significantly in the overall patient population when the examination 1–14 days and ≥1 month postoperatively were compared (*p* = 0.021). In five of eight eyes (62.5%) with intraoperatively detected retinal folds, RD was evident on the examination 1–14 days postoperatively. However, there was no significant correlation between retinal detachment and intraoperatively detected retinal folds.

### 3.3. Postoperative Vision

On examination < 1 month postoperatively, 17/20 eyes (85%) in the NPC group and 8/12 eyes (67%) in the PC group were visual (*p* = 0.218) (total: 25/32 [78.1%]). During examination ≥1 month postoperatively, 14/18 eyes (77.8%) in the NPC group and 1/10 eyes (10%) in the PC group were visual (*p* < 0.001) (total 15/28 [53.6%]). 18/23 eyes (78.2%) and 7/9 eyes (77.7) were visual during the examination <1 month postoperatively in the ERU group and non-ERU group, respectively. During examination at ≥1 month postoperatively, 11/20 eyes (55%) in the ERU group and 4/8 eyes (50%) in the non-ERU group were visual. In the overall patient population, postoperative vision correlated significantly with retinal detachment on examinations < 1 month (phi = 0.908; *p* < 0.001) and ≥1 month (phi = 1.0; *p* < 0.001) postoperatively. Six out of seven blind eyes (85.7%) showed RD on examination < 1 month postoperatively, while the remaining one eye (14.3%) was blind due to diffuse corneal oedema. On examination ≥1 month postoperatively, all 13 blind eyes showed evidence for RD. A significant decrease in the number of visual eyes was observed in the overall patient population when comparing the six postoperative time periods (*p* < 0.001) (Table 5). The number of visual eyes was non-significantly higher in the NPC group at each of the six examination time points, and no obvious differences were observed between the aetiological groups (Table 5).

Based on binary logistic regression, the surgical technique significantly affected postoperative vision at the period < 1 month postoperatively, whereby vision was preserved in more eyes in the NPC group (OD 0.079; *p* = 0.048). In the subsequent five time periods (Table 5), the surgical technique had no significant influence on postoperative vision. No influence of the age of the horse or the aetiology of the cataract (ERU or non-ERU) on postoperative vision was identified at any of the six-time points.

## 4. Discussion

This study investigated the outcome of PE-PPV in horses with acquired cataracts associated with uveitis. The most important observations in the overall patient population were that no postoperative persistent active uveitis or ERU recurrence (in case of ERU-associated cataract) was observed during follow-up examinations. However, the proportion of eyes that were blind due to RD increased significantly over time postoperatively. Considering intraoperative as well as long-term complications in equine eyes undergoing PE-PPV with and without posterior capsulorhexis, both the rate of complications and the number of horses that subsequently lost vision were significantly higher in cases where posterior capsulorhexis were performed in this study. In contrast to this finding, no obvious differences were observed in the intraoperative and long-term complications rate between the aetiological groups.

Based on history and preoperative findings associated with previous uveitis in all cases, an increased risk of postoperative persistent active uveitis was assumed in the horses included in the current study [6,7,19]. To avoid additional postoperative ocular irritation, an intraocular lens was not applied in any case [26]. In line with previous reports, PE and PPV were combined in the present study to prevent postoperative persistent active uveitis [3,6,14]. Additionally, several studies propose PV as a measure for the prevention of retinal detachment in cases of uveitis-related vitreous degeneration, which was present in all cases of this study [13,14,25,27]. Another benefit of the PPV, in addition to the PE, is the improvement of vision through the removal of vitreal opacities, which were also present in all cases of this study [13]. To minimise the risk of general anaesthesia and trauma during recovery, PE and PPV were performed in one session [9].

Horses with intraoperative tearing of the posterior lens capsule, as well as advanced cataract stages with opacification of the lens capsule and/or degenerative adhesion between the lens capsule and cortex, required posterior capsulorhexis [9,28]. Despite the fact that the lens cortex/nucleus was removed before the posterior capsulorhexis (PC group), intraoperative migration of small lens remnants into the vitreous body/chamber occurred in 3/12 eyes. Intraoperative non-detection of small peripheral free cortex remnants is the most likely cause for the remnants that were evident postoperatively. Another possible explanation is the occurrence of small tears in the posterior lens capsule through which small lens fragments could dislodge the vitreous intraoperatively, which were not detected during surgery. Although the migration of lens remnants in the vitreous body/chamber could be associated with potentially severe concomitant complications [19], such complications were not observed in this study. The small size of the migrated fragments may most likely explain this outcome.

The presence of retinal folds was the most important intraoperative observation in this study. The fact that the PC group was affected more frequently may be related to more severe preoperative pathological findings. Additionally, posterior capsulorhexis and the resulting pressure changes in the anterior and vitreous chamber [29], as well as more invasive surgical steps [16], can trigger retinal fold formation. Due to the cataract, preoperative evaluation of the posterior ocular segment was limited to ultrasonography. It cannot be excluded that some preoperatively existing retinal folds remained undetected and were visible intraoperatively only following cataract removal. Hypotony caused by the removal of 2 mL of aqueous humour and/or placement of the intravitreal infusion approach for PPV before PE could potentially be another reason for the observed retinal folds. However, as the IOP was maintained at 25 mmHg throughout the surgery by the intravitreal infusion, the authors regard this scenario as highly unlikely. Retinal folds are reported to resolve with a return to physiological IOP [7]. Despite the recovery of physiological IOP, RD was diagnosed within the first two postoperative weeks in 5 out of 8 cases with intraoperatively observed retinal folds. In one case, the retinal folds remained visible. In a previous study [7], most horses presented with congenital cataracts, whereas the current study included only horses with uveitis-associated cataracts. The inflammatory component is associated with more complications following PE and may explain the different outcomes [6,8].

Eyes that required posterior capsulorhexis presented with more severe pathological findings preoperatively [17,30]. This may explain the higher incidence of intra- and postoperative complications in the PC group (Table 2 and Table 4). Additionally, posterior capsulorhexis with the resulting pressure changes in the anterior and vitreous chambers [29], anterior displacement of the vitreous body [15] and more invasive surgical steps [16] can trigger several complications [9,31].

**Figure 3:**
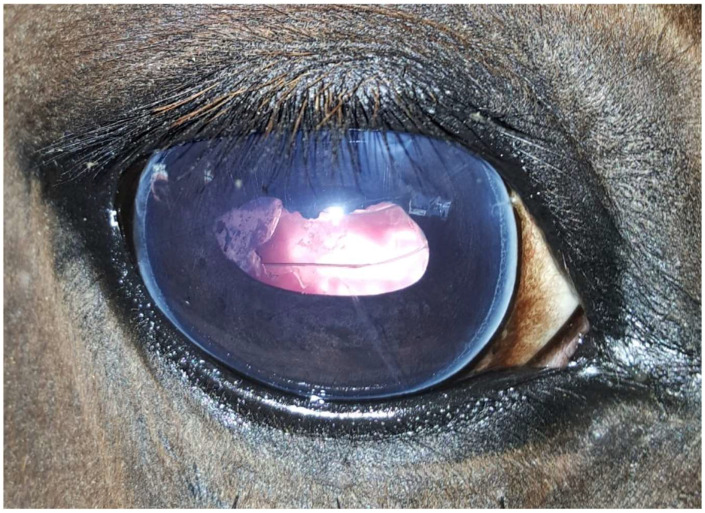
Same eye as in Figure 1 and Figure 2, ten months after phacoemulsification combined with pars plana vitrectomy without signs of uveitis. Moderate corneal fibrosis is visible in the approach region (one o’clock position), with mild opacification of the lens capsule caused by the regenerated lens epithelial cells at the nine o’clock position.

In the present study, the number of visual eyes correlated significantly with RD at all time points and decreased significantly with increasing time post-surgery. In previous reports, PCO in the posterior lens capsule, glaucoma and corneal oedema are described to be frequent causes of postoperative blindness following PE [6,7,8]. However, postoperative opacities in the remaining lens capsule were mild and did not significantly impair vision in the cases included in the present study (Figure 3). Only three eyes with an ERU-associated cataract developed PE-associated postoperative hypertension, most likely due to viscoelastic retention [7,8], which was resolved with medical management. Six out of seven blind eyes (85.7%) showed RD on examination < 1 month postoperatively, while only one eye (14.3%) was blind due to diffuse corneal oedema. On examination ≥1 month postoperatively, all 13 blind eyes showed evidence for RD. Previously reported rates of postoperative RD are lower, ranging 6.4–25% [6,8]. The differences may be explained by varying surgical techniques and intraoperative complications, perioperative medical management, cataract aetiology (more preoperative pathological findings) and study design [7,19,32,33]. The evaluation of the postoperative course in the present study was performed by a veterinarian in all cases, whereas the postoperative outcome was partly based on telephone interviews with the owners in previous reports [6,7,8]. 

Only horses with uveitis-associated cataracts were included in the present study. In comparable studies, only 13% [8] and 21.6% [7] of eyes were affected by uveitis-associated cataracts. Vitreous degeneration without active uveitis was detected in all eyes in the current report (see Table 2 and Table 4). Additionally, moderate preoperative, ultrasonographically undetectable retinal changes cannot be excluded. Both uveitis-induced conditions are a reported cause of RD following PE [6,14,34,35]. Based on binary logistic regression, vision in the first-month post-surgery was significantly influenced by the surgical technique. The one-factorial analysis identified a higher number of visual eyes and, therefore, lower RD incidence in the NPC group at all time periods. However, none of the differences was significant (Table 5), and the comparison was affected by several pre- and intraoperative factors. Whilst the authors considered posterior capsulorhexis and associated complications as the main reason for a guarded outcome, small animal studies also indicate that advanced stages of cataract decrease postoperative outcomes [36,37]. To prevent RD, laser-retinopexy and the temporary replacement of the vitreous with gas or silicone oil appear promising, but the techniques have only been validated in small animal studies so far [38,39].

In human ophthalmology and in a recent equine study, the loss of positional support of the retina by the vitreous body after PE-PPV has been described as increasing the risk for RD [5,29]. Since the eyes in the present study showed a certain degree of preoperative vitreous degeneration, suggesting that the positional support of the retina had already been weakened, PPV is unlikely to be the sole cause of RD [13,25]. Some equine studies describe the accidental contact of the probe with the retina during PPV to result in RD [5,18]. Inadvertent contact was, however, not observed during PPV in the present report.

The number of eyes with postoperative active uveitis decreased significantly when compared to the postsurgical and follow-up examinations despite the uveitis history in each case in the present study. Additionally, no postsurgical episodes of uveitis recurrence were reported by the owners. This contradicts several previous studies that suggested a high incidence of persistent active uveitis after PE for eyes with previous uveitis [6,7,8]. The different observations may be explained by the PPV and subconjunctival dexamethasone injection with consistent topical dexamethasone treatment performed in each case, which reduces the occurrence of uveitis [5,10,25].

Eyes with preoperatively detected posterior synechia occurred significantly more often in the ERU group. This could be associated with the history of recurrent inflammatory episodes, with a minimum of two episodes reported in the ERU group, whereas only one inflammatory episode was reported in the non-ERU group. However, no obvious differences were observed between the aetiological groups in intraoperative and postoperative outcomes. This could be explained by the uveitis-associated aetiology of cataracts as well as similar surgical and postsurgical treatment protocols in all cases of this study.

The decision to perform the posterior capsulorhexis was made intraoperatively, and the PC group included more eyes with severe preoperative pathological findings. This fact could impact the direct comparison of both surgical methods regarding the intra- and postoperative outcome. Further, it is not possible to differentiate between complications related to PPV and/or PE based on the present results. A standardised population and a control group without PPV would be necessary for an independent evaluation. However, due to the rarity of PE in horses, this attempt appears problematic under clinical conditions.

Additionally, the relatively high heterogeneity of the patient population and the limited sample size are limitations of the current study. The absence of preoperative electroretinograms is a further disadvantage; however, ophthalmic and ultrasonographic examinations did not identify any abnormalities of the retinal position in the overall patient population. A concern of this and previous studies is the method of postoperative visual evaluation [6,7,8,16]. Total visual loss was detected, but partial visual deficits were potentially missed.

## 5. Conclusions

Despite postsurgical uveitis immediately after PE-PPV, in some cases, no persistent active uveitis or ERU recurrence (in the case of ERU-associated cataracts) was observed in the present study during the follow-up examinations. However, the proportion of eyes that were blind due to RD increased significantly postoperatively. Whilst PE-PPV may prevent postsurgical persistent active uveitis and remove lens opacity, the prognosis for a visual outcome is guarded. Based on the results of this study, there is a tendency to prefer the NPC technique and give a more guarded prognosis for the PC technique. However, caution is required when interpreting these results due to several factors that affect the independent comparison of the two techniques in this study. Additional techniques for preventing postoperative RD and further refinement of the surgical technique, such as the development of laser-retinopexy in equine eyes, are required to continuously improve postoperative vision long-term.

## Figures and Tables

**Table 1:** Distribution of horse breeds and sex in the overall patient population, the surgical and aetiological groups.

	Overall *n* = 28	NPC *n* = 18	PC *n* = 10	ERU *n* = 21	Non-ERU *n* = 7
Breed					
Warmblood	11	8	3	7	4
Appaloosa	1	1		1	
German Riding Pony	5	1	4	4	1
Icelandic horse	6	4	2	6	
Polo horse	1		1	1	
Gypsy horse	1	1			1
Standardbred	2	2		1	1
Quarter Horse	1	1		1	
Sex					
Stallions	5	3	2	4	1
Mares	10	7	3	6	4
Geldings	13	8	5	11	2

NPC—no posterior capsulorhexis group; PC—with posterior capsulorhexis group; ERU—equine recurrent uveitis.

**Table 2:** Cataract characteristics and intraoperative findings in the overall patient population, the surgical and aetiological groups. For maturity differentiation, cataracts were divided into immature, mature and hypermature.

	Overall (%) *n* = 32	NPC (%) *n* = 20	PC (%) *n* = 12	ERU (%) *n* = 23	Non-ERU (%) *n* = 9
Cataract maturity					
Immature	8 (25)	5 (25)	3 (25)	6 (26)	2 (22.2)
Mature	20 (62.5)	15 (75)	5 (41.7)	14 (60.8)	6 (67)
Hypermature	4 (12.5)		4 (33.3)	3 (13)	1 (11)
Cataract localization					
Cortex, Nucleus	25 (78.1)	20 (100)	5 (41.7)	18 (78.2)	7 (77.7)
Capsule, Cortex, Nucleus	7 (21.9)	0 (0)	7 (58.3)	5 (21.7)	2 (22.2)
Intraoperative findings					
Posterior lens capsule tear	5 (15.6)	0 (0)	5 (41.7)	4 (17.4)	1(11)
Retinal folds	8 (25)	1 (5)	7 (58.3)	6 (26)	2 (22.2)
Retinal detachment	2 (6.3)	0 (0)	2 (17)	1 (4.3)	1 (11.1)
Vitreous haemorrhage	3 (9.4)	0 (0)	3 (25)	2 (8.7)	1 (11.1)
Vitreous debris/degeneration	32 (100)	20 (100)	12 (100)	23 (100)	9 (100)
Lens remnants displacement into the vitreous body	3 (9.4)	0 (0)	3 (25)	2 (8.7)	1 (11.1)

NPC—no posterior capsulorhexis group; PC—with posterior capsulorhexis group; ERU—Equine recurrent uveitis.

**Table 3:** Ophthalmic examination findings of surgically treated eyes during three different time periods, in the overall patient population, the surgical and aetiological groups.

	Preoperative					1–14 Days PO					≥1 Month PO				
	Overall (%)	NPC (%)	PC (%)	ERU (%)	Non-ERU (%)	Overall (%)	NPC (%)	PC (%)	ERU (%)	Non-ERU (%)	Overall (%)	NPC (%)	PC (%)	ERU (%)	Non-ERU (%)
Diffuse corneal oedema	0/32 (0)	0/20 (0)	0/12 (0)	0/23 (0)	0/9 (0)	6/32 (19)	2/20 (10)	4/12 (33.3)	5/23 (21.7)	1/9 (11.1)	2/28 (7.1)	0/18 (0)	2/10 (20)	2/20 (10)	0/8 (0)
Posterior synechia	17/32 (53)	10/20 (50)	7/12 (58)	15/23 (65.2)	2/9 (22.2)	12/30 (40)	8/18 (44.4)	4/12 (33.3)	10/21 (47.6)	2/9 (22.2)	14/26 (53.8)	10/17 (58.8)	4/9 (44.4)	11/18 (61.1)	3/8 (37.5)
Dyscoria	10/32 (31)	5/20 (25)	5/12 (41.7)	8/23 (34.8)	2/9 (22.2)	10/31 (32.3)	6/19 (31.6)	4/12 (33.3)	8/22 (36.3)	2/9 (22.2)	13/26 (50)	9/17 (52.9)	4/9 (44.4)	10/18 (55.5)	3/8 (37.5)
PCO in the posterior lens capsule	N/A	N/A	N/A	N/A	N/A	2/19 (10.5)	2/19 (10.5)	N/A	2/14 (28.5)	0/5 (0)	7/18 (38.8)	7/18 (38.8)	N/A	5/12 (41.7)	2/6 (33.3)
Postoperative lens remnants in the vitreous cavity	N/A	N/A	N/A	N/A	N/A	3/25 (12)	0/16 (0)	3/9 (33.3)	2/19 (10.5)	1/6 (16.7)	1/20 (5)	0/13 (0)	1/7 (14.3)	1/14 (7.1)	0/6 (0)
Retinal detachment	0/32 (0)	0/20 (0)	0/12 (0)	0/23 (0)	0/9 (0)	6/32 (18.8)	2/20 (10)	4/12 (33.3)	4/23 (17.4)	2/9 (22.2)	13/28 (46.4)	4/18 (22.2)	9/10 (90)	9/20 (45)	4/8 (50)
Retinal fold formation	N/A	N/A	N/A	N/A	N/A	1/32 (3.1)	0/20 (0)	1/12 (8.3)	1/23 (4.3)	0/9 (0)	1/28 (3.6)	1/18 (5.6)	0/10 (0)	1/20 (5)	0/8 (0)
Retinal oedema	N/A	N/A	N/A	N/A	N/A	3/32 (9.4)	1/20 (5)	2/12 (16.7)	2/23 (8.7)	1/9 (11.1)	1/28 (3.6)	1/18 (5.6)	0/10 (0)	1/20 (5)	0/8 (0)
Postoperative hypertension	0/32 (0)	0/20 (0)	0/12 (0)	0/23 (0)	0/9 (0)	3/32 (9.4)	1/20 (5)	2/12 (16.7)	3/23 (13)	0/9 (0)	0/28 (0)	0/18 (0)	0/10 (0)	0/20 (0)	0/8 (0)

NPC—no posterior capsulorhexis group; PC—with posterior capsulorhexis group; ERU—equine recurrent uveitis; PCO—postoperative capsular opacification; PO—postoperative; N/A = not evaluated.

**Table 4:** Ophthalmic examination findings are associated with uveitis of surgically treated eyes during three different time periods in the overall patient population and the surgical and aetiological groups.

	Preoperative					1–14 Days PO					≥1 Month PO				
	Overall (%)	NPC (%)	PC (%)	ERU (%)	Non-ERU (%)	Overall (%)	NPC (%)	PC (%)	ERU (%)	Non-ERU (%)	Overall (%)	NPC (%)	PC (%)	ERU (%)	Non-ERU (%)
AC-flare score (0; 1; 2–3) ^a^															
0	31/32 (100)	19/20 (100)	12/12 (100)	23/23 (100)	9/9 (100)	18/31 (58)	15/19 (79)	3/12 (25)	12/22 (54.5)	6/9 (67)	27/27 (100)	18/18 (100)	9/9 (100)	19/19 (100)	8/8 (100)
1	0/32 (0)	0/20 (0)	0/12 (0)	0/23 (0)	0/9 (0)	6/31 (19.3)	2/19 (10.5)	4/12 (33.3)	5/22 (22.7)	1/9 (11.1)	0/27 (0)	0/18 (0)	0/9 (0)	0/19 (0)	0/8 (0)
2–3	0/32 (0)	0/20 (0)	0/12 (0)	0/23 (0)	0/9 (0)	7/31 (22.7)	2/19 (10.5)	5/12 (41.7)	5/22 (22.7)	2/9 (22.2)	0/27 (0)	0/18 (0)	0/9 (0)	0/19 (0)	0/8 (0)
Vitreous chamber haze score (0; 1; 2; 3) ^a^															
0	N/A	N/A	N/A	N/A	N/A	9/26 (34.6)	7/17 (41.2)	2/9 (22.2)	6/19 (31.5)	3/7 (42.8)	22/23 (95.7)	15/16 (93.8)	7/7 (100)	16/17 (94.2)	6/6 (100)
1	N/A	N/A	N/A	N/A	N/A	7/26 (27)	5/17 (29.4)	2/9 (22.2)	5/19 (26.3)	2/7 (28.6)	1/23 (4.3)	1/16 (6.2)	0/7(0)	1/17 (5.8)	0/6 (0)
2	N/A	N/A	N/A	N/A	N/A	9/26 (34.6)	5/17 (29.4)	4/9 (44.4)	7/19 (36.8)	2/7 (28.6)	0/23 (0)	0/16 (0)	0/7(0)	0/17 (0)	0/6 (0)
3	N/A	N/A	N/A	N/A	N/A	1/26 (3.8)	0/17 (0)	1/9 (11.2)	1/19 (5.2)	0/7 (0)	0/23 (0)	0/16 (0)	0/7(0)	0/17 (0)	0/6 (0)
Hyperechoic material in vitreous chamber ^b^	21/32 (65.5)	13/20 (65)	8/12 (68)	16/23 (70)	5/9 (55.5)	8/26 (30.7)	6/17 (35.3)	2/9 (22.2)	6/20 (30)	2/6 (33.3)	0/21 (0)	0/14 (0)	0/7 (0)	0/15 (0)	0/6 (0)
Active uveitis ^c^	0/32 (0)	0/20(0)	0/12 (0)	0/23 (0)	0/9 (0)	24/32 (75)	13/20 (65)	11/12 (91.7)	18/23 (78)	6/9 (67)	0/28 (0)	0/18 (0)	0/10 (0)	0/20 (0)	0/8 (0)

^a^ Uveitis-associated ophthalmic findings were scored in surgically treated eyes according to the SPOTS Ocular Scoring System [22]. ^b^ Hyperechoic material was detected ultrasonographically. ^c^ Based on the uveitis-associated ophthalmic and ultrasonographic findings the diagnosis of active uveitis was given. NPC—no posterior capsulorhexis group; PC—with posterior capsulorhexis group; ERU—equine recurrent uveitis; PO—postoperative; AC—anterior chamber; N/A = not evaluated.

**Table 5:** Number of visual eyes following surgical treatment during seven different time periods in the overall patient population, the surgical and aetiological groups.

	Overall (%)	NPC (%)	PC (%)	ERU (%)	Non-ERU (%)
Preoperative	0/32 (0)	0/20 (0)	0/12 (0)	0/23 (0)	0/9 (0)
<1 month PO	25/32 (78.1)	17/20 (85)	8/12 (67)	18/23 (78.2)	7/9 (77.7)
1–6 months PO	23/32 (72)	16/20 (80)	7/12 (58.3)	16/23 (69)	7/9 (77.7)
6–12 months PO	10/19 (52.6)	7/11 (63.6)	3/8 (37.5)	7/12 (58.3)	3/7 (42.8)
12–18 months PO	5/16 (31.3)	3/7 (42.9)	2/9 (22.2)	3/10 (30)	2/6 (33.3)
18–24 months PO	4/16 (25)	3/8 (37.5)	1/8 (12.5)	2/9 (22.2)	2/7 (28.5)
>24 months PO	3/15 (20)	2/7 (28.6)	1/8 (12.5)	2/10 (20)	1/5 (20)

NPC—no posterior capsulorhexis group; PC—with posterior capsulorhexis group; ERU—equine recurrent uveitis; PO—postoperative.

## Data Availability

The data presented in this study are available on request from the corresponding author. The data are not publicly available due to privacy issues.
